# Fabrication and Characterization of Alveolus-Like Scaffolds with Control of the Pore Architecture and Gas Permeability

**DOI:** 10.1155/2022/3437073

**Published:** 2022-01-20

**Authors:** Lanxin Lü, Hongxian Shen, Daichi Kasai, Ying Yang

**Affiliations:** ^1^Emergency Center of the Affiliated Hospital of Xuzhou Medical University, Laboratory of Emergency Medicine, Xuzhou Medical University, Xuzhou 221002, China; ^2^School of Pharmacy and Bioengineering, Keele University, Stoke-on-Trent ST4 7QB, UK; ^3^School of Life Sciences, Guangzhou University, Guangzhou, China

## Abstract

The micrometer scale sac-like alveoli are the most important and essential unit for gas exchange in the lung. Thus, design and fabrication of scaffolds for alveoli regeneration by tissue engineering approach should meet a few topography and functional requests such as large surface area, flexibility, and high gas permeability to their native counterpart. Testing the gas permeability of scaffolds through a fast and simple technique is also highly demanded to assist new scaffold development. This study fabricated alveolus-like scaffolds with regular pore shape, high pore connectivity, and high porosity produced by inverse opal technique alongside randomly distrusted porous scaffolds by salt leaching technique from two different materials (polyurethane and poly(L-lactic acid)). The scaffold surface was modified by immobilization of VEGF. A facile and new technique based on the bubble meter principle enabling to measure the gas permeability of porous scaffolds conveniently has been developed specifically. The cellular response of the scaffolds was assessed by culturing with bone marrow mesenchymal stem cells and coculturing with lung epithelial NL20 and endothelial HUVECs. Our results showed that the newly designed gas permeability device provided rapid, nondestructive, reproducible, and accurate assessment of gas permeability of different scaffolds. The porous polyurethane scaffolds made by inverse opal method had much better gas permeability than other scaffolds used in this study. The cellular work indicated that with VEGF surface modification, polyurethane inverse opal scaffolds induced alveolus-like tissues and have promising application in lung tissue engineering.

## 1. Introduction

The prevalence of lung diseases has been increasing because of smoking, air pollution (high dust and chemical particles in air), and genetic disorders [1]. Chronic obstructive pulmonary disorder (COPD), acute lung injury/acute respiratory distress syndrome (ALI/ARDS), pulmonary hypertension (PH), cystic fibrosis (CF), and lung cancer are among the lung diseases with high mortality [2–6]. However, human lungs have very limited capacity to regenerate. Once the lung is subjected to damage or degeneration, the function of the lung will undergo irreversible impairment. Lung tissue transplantation is the gold standard for treating damaged lungs and helping to save lives. It is an effective and safe therapy for the patients suffering from a variety of end-stage pulmonary diseases. Meanwhile, more than 50,000 adult lung transplants have been entered into the International Society of Heart and Lung Transplantation Registry by 2014 [7]. However, this treatment faces big challenges: shortage of donor organs, expensive surgery, and short transplant life due to chronic lung allograft dysfunction, along with the recurrence of the underlying pathology in some cases [8]. The late mortality following lung transplantation presents in approximately 50% of patients at 5 years after transplantation [7]. Therefore, tissue engineering approach which is able to generate biologically compatible substitutes to restore and support lung tissue functions [9] becomes a very promising therapy.

Good gas permeability is the key feature of the lung. Alveoli are the basic and essential unit of gas exchange. Scaffolds for alveoli regeneration through tissue engineering approach should have multiple connective pores and excellent gas permeability in the pore wall. Many scaffolds, such as decellularized, Gelfoam sponge, and collagen microcapsule [10–12], have been explored to fabricate tissue engineering lung with growing lung cells such as epithelial and endothelial cells in them. Previously, inverse opal scaffolds with uniform and controllable pore size, high pore connectivity, and thin pore wall were employed to investigate the blood vessel infiltration [13]. The structural features of inverse opal scaffolds can mimic topographic alveoli of the lung and make itself a promising scaffold candidate for lung tissue engineering.

To characterize whether a scaffold is suitable for alveoli production, gas permeability tests should be undertaken. A few reliable gas permeability testers for food packaging, textiles, and papers are available based on the manometer principle and ASTM standards, for example, the Permeance Testing Device, type GDP-C; Differential Pressure Air Permeability tester, Frazier; and ASTM 1434-82 and ASTM D737 [14, 15]. However, they are not suitable for testing the gas permeability of porous scaffolds in tissue engineering due to their small bulk size, relatively large pore, and unique architecture [16, 17]. Considering the high gas permeability of porous scaffolds, the high-precision flowmeter of gas is needed in gas measurement of the test. Levy reported the bubble meter technique as a simple but reliable method of measuring the volume flow rate of gases in application [18]. The aim of the current study is to fabricate and compare multiple alveolus-like scaffolds used for lung tissue engineering and design a convenient and facile device capable of testing the gas permeability of these produced scaffolds in order to guide better fabrication of alveolus-like scaffolds.

## 2. Materials and Method

### 2.1. Fabrication of Alveolus-Like Scaffolds

Two types of porous scaffolds with different architectures were fabricated. One type was gelatin-based inverse opal scaffolds with uniform pore structures, denoted as IOS, and the other one was salt leaching porous scaffolds with randomly distributed pore structures, denoted as SLS. Two types of polymers, polyurethane (PU) (Sigma-Aldrich, US) and poly(L-lactic acid) (PLLA) (Purac BV, Gorinchem, the Netherlands) have been used to fabricate the scaffolds.

**Figure 1:**
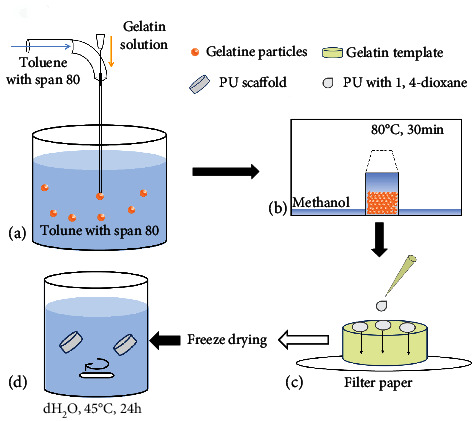
The schematic of 3D porous scaffold fabrication by inverse opal method. (a) Gelatin particle generation by the microfluidic device; (b) gelatin template formation in methanol atmosphere at a 80°C oven for 30 minutes; (c) PU solution penetration through gelatin template and filling the gaps between each gelatin particle; (d) PU scaffold collection after freeze drying for 6 hours and removal of gelatin particles by immersing in a water bath at 45°C for 24 hours.

IOS scaffolds were generated following the established protocol with slight modification as shown in Figure 1 [13]. Firstly, uniform gelatin microspheres with sizes ranging from 180 *μ*m to 430 *μ*m through altering experimental parameters (flow rate of toluene and gelatin solution and diameter of capillary) (Table 1) were produced using a microfluidic device shown in Figure 1(a). Secondly, the template moulds with multilayered hexagonally packed gelatin microspheres were formed (Figure 1(b)). Then 15% (*w*/*v*) PU solution in 1,4-dioxane (Sigma-Aldrich, US) was dropped into the moulds (Figure 1(c)). The compounded moulds were frozen at -20°C for 6 hours and then lyophilized overnight in a freeze-dryer to remove the solvent. Finally, the gelatin microspheres were removed through immersing the moulds in a water bath at 43°C with gentle stirring overnight (Figure 1(d)). PU-IOS scaffolds with 5 mm height and 5 mm diameter were obtained.

To generate SLS scaffolds, sodium chloride (NaCl) particles with the diameter between 250 *μ*m and 350 *μ*m were collected by sieves and then mixed with 15% PU solution or 6% (*w*/*v*) PLLA solution (in chloroform, Sigma, UK) for PU and PLLA scaffolds, respectively, with the weight ratio of polymer solution and NaCl at 1 : 9. The salt and polymer composites were kept in a cylinder-shaped mould for 3-5 days to evaporate the organic solvent, after which the composites were immersed in a water bath to leach out the salt particles thoroughly. The SLS scaffolds with diameter of 5 mm and height of 5 mm were collected.

### 2.2. Device Design for Gas Permeability Test

**Figure 2:**
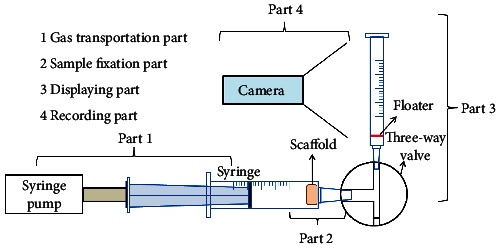
Schematic of designed gas permeability test device with four parts. 1: gas transportation part composed of a syringe pump and a syringe; 2: sample fixation part, enabling samples against the vent stably; 3: displaying part which uses a detergent film as the gas floater; 4: recording part composed of a digital camera.

The convenient and simple device to assess gas permeability of porous scaffolds based on the bubble meter principle was designed [18, 19]. The device consisted of 4 parts as schematically drawn in Figure 2. The part 1 was used for gas transportation; it was composed a syringe pump (KD Scientific) and a 50 mL syringe (Norm Ject). The part 2 was utilized for sample holding. The porous scaffolds with defined dimension and a larger surface area than that of the vent of the 50 mL syringe were placed against the vent stably. The part 3 was the displaying component consisting of a flow direction changer via a three-way adaptor, a 2 mL syringe (Norm Ject) without plunger, and a floater which was made by the detergent film generated from a mixture viscous solution of detergent and distilled H_2_O at the ratio of 4 : 1. A digital camera recorded the floater movement as the part 4. After the detergent film was placed near the bottom of the 2 mL syringe, the syringe pump was set up to push the air inside the syringe through the scaffold by various flow rates. The movement rate of the floater was used as the readout of the different types of gas permeability of the scaffolds.

### 2.3. Characterization of Alveolus-Like Scaffolds

#### 2.3.1. Observation of Scaffolds

The morphology of IOS and SLS scaffolds made by PU and PLLA, gelatin microspheres, and hexagonally packed gelatin moulds was observed by bright field microscope (Olympus, US) and SEM (FEI Teneo VS, US).

#### 2.3.2. Gas Permeability

The gas permeability of PU-IOS scaffolds with various pore sizes and SLS scaffolds made from PLLA and PU was measured by the designed device. Firstly, the scaffolds (*ϕ* 5 × 5 mm) were placed inside the 50 mL syringe with stable attachment. The flow rate of the syringe pump was set up as 5, 10, 20, 30, and 40 mL/min, for each sample. For each flow setting and test, the movement distances of floater indicating as the initial height, *H*_0__,_ and the final height, *H*_1__,_ corresponding to initial time, *T*_0__,_ and final time, *T*_1__,_ respectively, were recorded by the camera. The movement rate of the floater (*v*) was calculated by the following formula:

(1) $$\begin{array}{c} v=\frac{H_{1}-H_{0}}{T_{1}-T_{0}}. \end{array}$$

Each experiment was repeated at least three times. The gas permeability between the scaffold samples was compared by the floater movement rate.

#### 2.3.3. Mechanical Property

The mechanical property of IOS and SLS scaffolds (*ϕ* 5 × 5 mm) was measured by mechanical testing machine (BOSE Electro Force 3200 Series, UK) at a speed of 10 mm/min under the compression mode. The stress and strain were plotted from original force-deformation curves according to the following formulas. Young's modulus was calculated accordingly.

(2) $$\begin{array}{c} \text{Stress}=\frac{\text{Force applied}}{\text{Area of specimen surface}},\\ \text{Strain}=\frac{\text{Original height}-\text{Final height}}{\text{Original height}},\\ \text{Youn}{\text{g}}^{\text{'}}\text{s modulus}=\frac{\text{Stress}}{\text{Strain}}. \end{array}$$

### 2.4. Fabrication of Tissue-Engineered Alveoli

#### 2.4.1. Surface Modification

In order to enhance the cell attachment to PU-IOS, surface modification was conducted through introduction of vascular endothelial growth factor (VEGF) to scaffolds covalently [20]. Firstly, ammonia plasma treatment was applied to introduce the amine group to PU scaffolds. To do so, PU scaffolds were put into the plasma reaction chamber whose pressure was increased from 21 mbar to 50 mbar with ammonia gas pumping. The plasma treatment lasted for 5 minutes. Scaffolds were immersed into 70% ethanol for 2 hours for sterilization. Secondly, heparin was introduced onto the scaffold surface through crosslinking reaction with 1-ethyl-3-(3-dimethylaminopropyl) carbodiimide hydrochloride (EDC) and N-hydroxysulfosuccinimide (Sulfo-NHS). Ammonia plasma-treated PU scaffolds were immersed into the solution of EDC (2 mM), Sulfo-NHS (5 mM), and heparin (1 mg/mL) in 2-(morpholino)ethanesulfonic acid (MES) buffer and incubated for 2 hours at room temperature to bind heparin. After washing three times with PBS, the scaffolds were immersed into 100 *μ*L of VEGF solution (100 ng/mL) for grafting at 4°C overnight. The grafted scaffolds were washed with PBS and kept at 4°C for further application.

#### 2.4.2. MSCs Culture

MSCs isolated from rat bone marrow as described in our previous study [21] were used in this study. In brief, bone marrow in tibias and femurs from 4-week-old Sprague−Dawley rats was flushed with 10 mL of cell culture medium and transferred into two T25 culture flask (NUNC, US) and incubated by *α*-MEM medium (Hyclone, USA) supplemented with 10% fetal bovine serum (FBS, BI, Israel) and 1% antibiotics (Hyclone, USA) at 37°C with 5% CO_2_. The medium was replaced every 3 days until cells reached confluence. Cells were subcultured at a 1 : 2 plate ratio. Passage 3-5 MSCs were seeded onto the VEGF-modified alveolus-mimicking porous scaffolds with cell seeding density of 10^4^/mm^3^. Neat PU-IOS scaffolds seeded with MSCs were used as the control group. The constructs were cultured by the above-mentioned *α*-MEM medium at 37°C with 5% CO_2_, and the medium was changed every two days.

#### 2.4.3. Coculture

Human umbilical vein endothelial cells (HUVECs, Lonza) and human bronchial epithelial cell lines (NL20, ATCC) were cocultured to observe the localization of the two types of cells in the alveolus-like scaffolds. The media for NL20 contained Ham's F12 (Lonza, UK), with 4% FBS, 1.3% of 1.5 g/L NaHCO_3_, 1% of 2 mM L-glutamine, 1% of 0.1 mM nonessential amino acids, 1% of antibiotic antimitotic, and 0.05% of 0.5 *μ*g/mL hydrocortisone. The media for HUVECs was composed of Media 200 (M200, Thermo Fisher) with 2% low serum growth supplement (LGSS, Thermo Fisher). The NL20 and HUVECs with the ratio of 4 : 1 and cell density of 10^5^/mm^3^ were seeded onto scaffolds with a dimension of 50 mm^3^. NL20 were labelled with the PKH26 cell linker (Red, Sigma-Aldrich) following the product instruction before cell seeding, and HUVECs were identified by staining with CD31 after termination of the culturing. The cell-scaffold constructs were cultured in a mixed medium (with the ratio of 1 : 1), and the medium was changed every 2 days.

### 2.5. Characterization of Tissue-Engineered Alveoli

#### 2.5.1. CCK-8 Assay

The viability and proliferation of MSCs on VEGF modified and nonmodified PU-IOS scaffolds were tested by cell counting kit-8 (CCK-8, Dojindo, Japan) as described in our previous study [20]. Briefly, at 1, 3, and 5 days after cell seeding, CCK-8 solutions at a dilution of 1 : 10 with media were added to each sample and cultured at 37°C with 5% CO_2_. Two hours later, 100 *μ*L of media were transferred into a 96-well plate to measure the absorption value at a wavelength of 450 nm using a microplate reader (Symergy HT, BioTek, USA).

#### 2.5.2. Confocal Observation of Cell Viability and Morphology

The viability of MSCs cultured on VEGF modified and nonmodified PU scaffolds at day 7 was determined by live and dead staining assay according to the product manual (Viability/Cytotoxicity Kit, Invitrogen, USA). Confocal microscopy (Leica, Germany) was used to observe the live and dead MSCs on PU scaffolds. Live cells were imaged as green color at an excitation of 488 nm and dead cells as red at an excitation of 535 nm. The three-dimensional (3D) images were reconstructed by *z*-stacking two-dimensional images with 10 *μ*m steps.

After 21 days of coculturing, the location and distribution of NL20 and HUVECs within the scaffolds were examined by expressed fluorescence; NL20 was displayed as red color by labelling with the PKH26 cell linker dye, whilst the HUVECs were stained with CD31 antibody. To stain the CD31 marker, the constructs were fixed by 4% paraformaldehyde (PFA) first at room temperature for 30 minutes. After removal of PFA, the constructs were washed with PBS twice, put into 1% BSA blocking solution, and incubated for one hour. After washing twice with PBS, the CD31 primary antibody (goat-antihuman, Santa Cruz, UK), diluted with 1% BSA with the ratio of 1 : 100, was added and incubated for at least 16 hours at 4°C. Constructs were washed with PBS three times and incubated with secondary antibody (FITC-donkey antigoat, 1 : 100, Santa Cruz, UK) at 37°C for 3 hours. Finally, constructs were washed 3 times with PBS and counterstained by DAPI for the nucleus, then observed by confocal microscopy.

### 2.6. Statistical Analysis

All quantitative results were expressed as mean ± standard deviation. At least 6 replicates were used for each group. Statistical analysis was carried out using one-way ANOVA and *t*-test for each two-group comparisons to determine significance. A value of *P* < 0.05 was considered to be statistically significant.

## 3. Results

**Figure 3:**
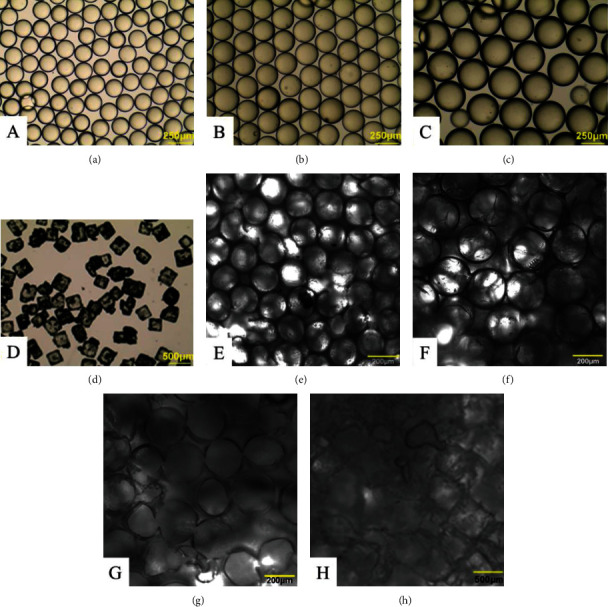
Visible images of porogens and the pores within scaffolds. Gelatin particles generated by the microfluidic device with different diameters (a–c), NaCl particles (d), and porous scaffolds (e–h). Gelatin particles with diameter of 180 ± 18 *μ*m (a), 250 ± 25 *μ*m (b), 310 ± 45 *μ*m (c), and NaCl particle with size of 250-350 *μ*m (d). Scaffolds produced from inverse opal technique (e–g) and from salt leaching method (h).

Figure 3 shows the images of porogen particles and formed pores in the scaffolds. It can been seen that the microfluidic device generated uniform gelatin microspheres with diameter of 180 ± 18, 250 ± 25, and 310 ± 45 *μ*m (Figures 3(a)–3(c)) compared with the sieving method (250-350 *μ*m, Figure 3(d)). Figures 3(e)–3(g) show the PU porous scaffolds with uniform pores, and Figure 3(h) shows the PLLA SLS scaffold with irregular pores.

**Figure 4:**
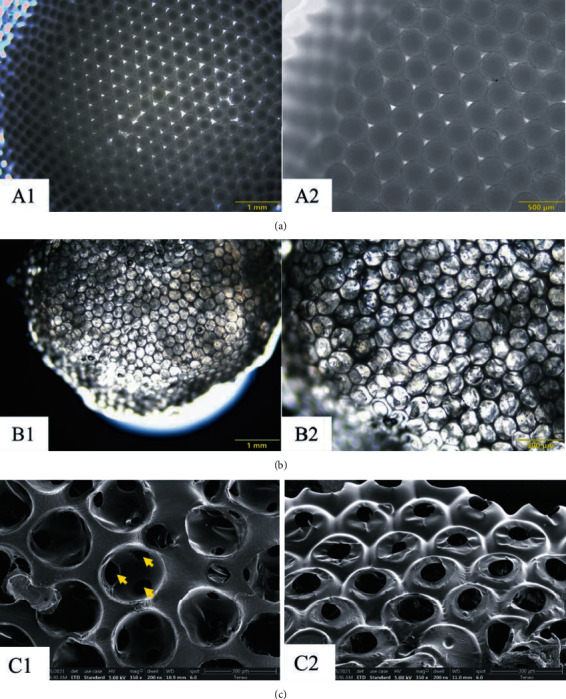
Images of gelatin microspheres packed moulds and formed scaffolds. Multilayered hexagonally packed gelatin microspheres (a) (A1, A2) and PU-IOS scaffolds (b) (B1, B2) from bright field microscopy. (c) (C1, C2) The formed PU scaffold observed by SEM. Arrows showed the smaller pores within big pores, which were created through connected gelatin microspheres.

Figure 4 shows the multilayered hexagonally packed gelatin microspheres (Figure 4(a) and PU-IOS scaffolds (Figures 4(b) and 4(c) used for MSC culturing. It can be seen that the gelatin microspheres we obtained had uniform diameters and the packed microspheres formed a hexagonal connection. Figure 4(C1) shows the cross-section of PU-IOS scaffolds with uniform pores and pore connection, and Figure 4(C2) shows the morphology of PU-IOS scaffolds with small pores inside big pores, indicating that the PU material did not cover the full surface of gelatin microspheres and tightly packed gelatin microspheres created the pore connections.

**Figure 5:**
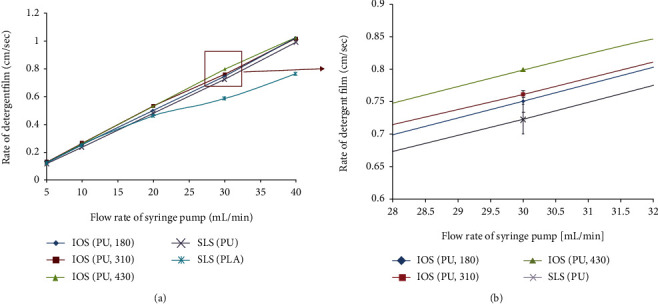
Gas permeability results of polyurethane scaffolds (IOS) made by inverse opal technique with different pore diameters and PLLA scaffolds (SLS) made from salt leaching method. (b) is the magnified image of the square part in (a).

The reliability of the gas permeability testing device we designed was assessed by analyzing the relationship of the gas flow rate and the floater movement rate. We tested the device without scaffolds by setting the flow rate of the syringe pump at 5, 10, 20, 30, and 40 mL/min and found that the detergent film as a floater of device went up at a constant rate under the corresponding pump rate condition and was reproducible (data not shown).  Figure 5 shows the results of gas permeability measurements of IOS and SLS scaffolds under different gas transportation rates. PU-IOS with pore size of 180, 310, and 430 *μ*m, PU-SLS, and PLLA-SLS with pore size of 250-350 *μ*m were used to validate the designed device. It can be seen from Figure 5 that there was no distinct difference between each groups when the gas transportation rate was 5, 10, and 20 mL/min. Whereas when the flow rate was increased to 30 mL/min, the gas permeability of PLLA-SLS was also obviously lower than the other groups, which indicated that PU had better gas permeability than PLLA. By closely monitoring the floater moving rate between 28 and 32 mL/min (Figure 5(b)), it was found that the PU-IOS had better gas permeability than PU-SLS. Furthermore, the gas permeability of PU-IOS increased gradually along with the increase of pore size. These results indicated that the type of material, pore morphology, and size contributed significantly to gas permeability.

**Figure 6:**
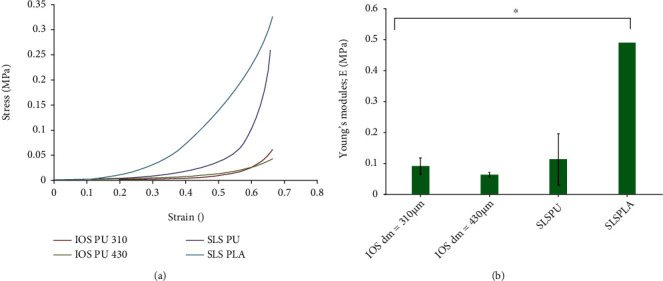
Mechanical property of polyurethane scaffolds made from inverse opal technique (IOS) and salt leaching method (SLS), and PLLA scaffolds made from salt leaching method (SLS). (a) Stress-Strain curves drawn according to compression data. (b) Young's modulus of scaffolds. *n* = 3; ^∗^*P* < 0.05.

The mechanical properties of PU-IOS and PU-SLS and PLLA-SLS are shown in Figure 6. It was clear that PU-IOS with a pore size of 310 and 430 *μ*m had similar stiffness, while the stiffness of PU and PLLA porous scaffolds produced by the salt leaching method was much higher than that of inverse opal scaffolds. Compared with PLLA, PU salt leaching-formed scaffolds were softer.  Figure 6(b) shows that PLLA-SLS scaffolds had the highest Young's modulus compared with PU scaffolds.

**Figure 7:**
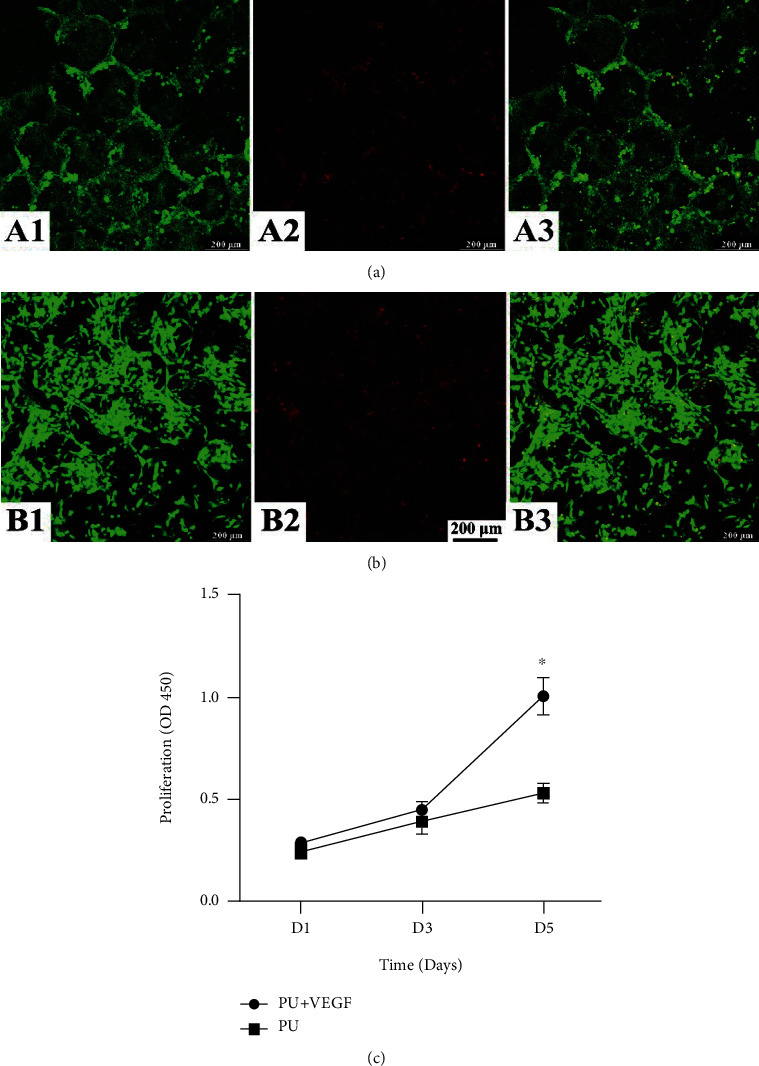
Confocal 2D images of the live and dead MSCs cultured in PU-IOS scaffolds for 7 days. (a) (A1–A3) Neat PU scaffolds; (b) (B1–B3) VEGF-modified PU-IOS scaffolds. Green color: live cells; red color: dead cells; (A3, B3) merged images. The quantitative cell viability cultured in the two types of scaffolds on days 1, 3, and 5 assessed by CCK-8 kit is presented in (c).

**Figure 8:**
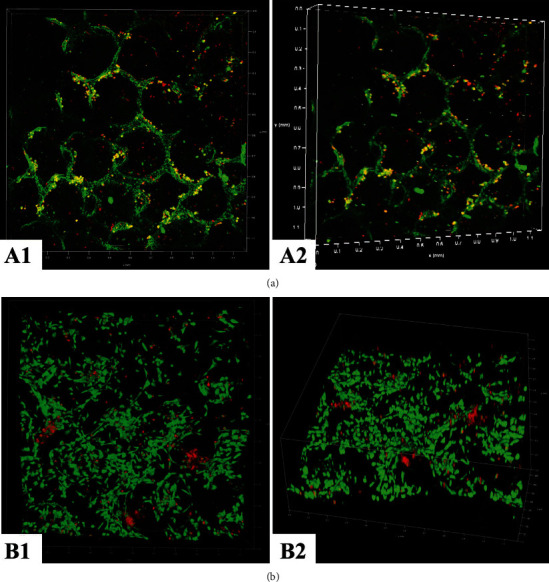
Confocal 3D images of the live and dead MSCs cultured in PU-IOS scaffolds for 7 days. (a) (A1, A2) Neat PU scaffolds; (b) (B1, B2) VEGF-modified PU IOS scaffolds; (A1, B1) top view; (A2, B2) side view. Green color: live cells; Red color: dead cells.

In Figures 7(a) and 7(b), the live and dead staining images show more live and less dead MSCs on VEGF-modified PU scaffolds Figure 7(b) than on neat PU scaffolds Figure 7(a) at day 7. Furthermore, it can be seen that MSCs spread well on VEGF-modified PU-IOS scaffolds instead of the round shape on PU neat scaffolds.  Figure 7(c) shows the CCK-8 results of MSCs cultured on PU-IOS scaffolds at days 1, 3, and 5. It is evident that MSCs on VEGF-modified PU-IOS scaffolds had better cell compatibility than those on unmodified PU scaffolds (*P* <0.05). From the 3D images, we can see that MSCs grew along the wall of the pores and formed alveolus-like structures on VEGF-modified PU-IOS scaffolds (Figure 8(b).

**Figure 9:**
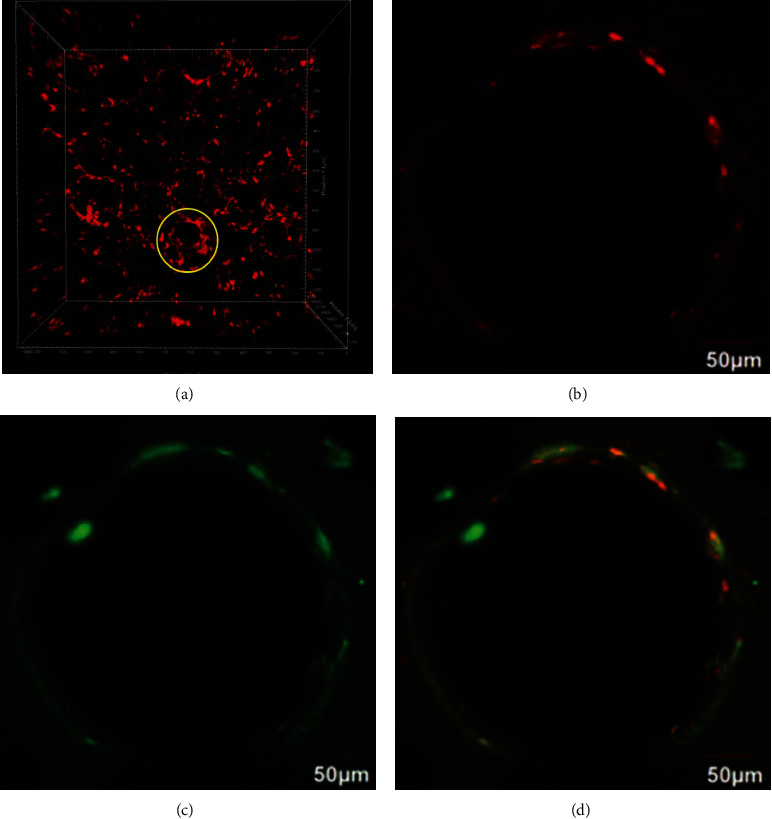
Confocal images of coculturing NL20 cells and HUVECs on VEGF modified PU-IOS scaffolds. NL20 cells were imaged live with labelling by PKH26 cell tracker (red color) on day 3 and day 21, whilst HUVECs were identified by CD31 staining (green color) on day 21. (a) Confocal live *z*-stacking 3D images of the construct cultured for 3 days. Yellow circle showed the cells attached onto the pore surface. (b) Confocal live 2D image of the construct cultured for 21 days; (c) confocal 2D image of the construct cultured for 21 days with CD31 staining showing the location of HUVECs cells; (d) merged images.

Figure 9 shows the NL20 attachment on day 3 and colocalization of NL20 and HUVECs on day 21 after being seeded. The red fluorescence in Figures 9(a) and 9(b) showed the NL20 cell tracking by PKH26 and the green color from the HUVEC stained by CD31 antibody (Figure 9(c)). In the merged image of Figure 9(d), we can see the yellow color which meant the colocalization of NL20 cells and HUVECs.

## 4. Discussions

Chronic lung diseases, such as COPD, CF, PH, and cancer, have no cure apart from lung transplantation. However, the critical shortage of donor lungs as well as the potential immunorejection limits the usage of lung transplantation. Generation of functional lung tissue through tissue engineering approach already demonstrates high promise to meet the clinical demands. Replication of the complex three-dimensional architectural structure and function of alveolar units becomes a challenge for successful tissue engineering of the lung. In this study, we adapted the inverse opal fabrication technique to produce porous scaffolds using highly elastic polyurethane (PU-IOS) in comparison to scaffolds made from the salt leaching technique by stiff polylactic acid with focus on the gas permeability and cellular attachment capacity between the scaffolds. PU-IOS scaffolds with VEGF modification generated consistent superior outcomes than other types of scaffolds. The new gas permeability test device demonstrated reliable measurement outcomes of various scaffolds.

Gelfoam as a hemostatic surgical material has been widely used in clinics for decades. As the temporal scaffolds, Gelfoam will be completely degraded or absorbed in vitro and in vivo quickly. Its porous structure and appropriate pore size similar to alveoli have been used in tissue engineering of the lung including us [11, 22–24]. Andrade et al. used Gelfoam sponge and fetal rat lung cells to regenerate an alveolus-like structure and explored the potential application for lung regeneration [11]. Gelfoam made from gelatin has low stability in an aqueous solution and enzyme environment. Compared with Gelfoam sponge, using the inverse opal fabrication technique can generate scaffolds with uniform and controllable pore size and regular pore connection, which mimics the alveolar cluster better and is therefore appropriate for lung tissue engineering.

Because of the elastic and permeability properties, PU and its family members have been used as an airway stent in bronchotracheal cancer and dynamic culture chamber in lung tissue engineering [25, 26]. To improve the biocompatibility, PU was modified by VEGF in this study, which can enhance the attachment and proliferation of MSCs. The results of CCK-8 at days 1, 3, and 5 and live and dead staining at day 7 showed that the modification of VEGF was very effective, which was also verified in our previous study [20]. Interestingly, when coculturing endothelial cells, HUVEC, with epithelial cells, NL20, colocalization of the two types of cells was clearly visible (Figure 9), which indicated that the inverse opal-fabricated scaffolds supported multiple cell populations on the alveolus-like structure.

We successfully grew MSC in PU-IOS scaffolds aiming to explore new cell sources for lung regeneration. It is clinically difficult to obtain autologous lung cells (endothelial, epithelial, fibroblasts) to treat patients with the aforementioned lung diseases. Induction and differentiation of MSC or lung progenitor cells [27] represent a promising and realistic direction to generate alveolar tissues by the lung tissue engineering approach. Figures 7 and 8 show that MSCs proliferated along with the wall of pores; it can still be found that MSCs cannot form a whole pore structure like alveoli, which was probably due to the short culture time enabling to form the cell sheet and also the static culture method. This is a limitation of our study. A longer culture time and dynamic culture method should be used to form an alveolar structure in future studies.

Gas permeability is one essential feature of lung tissue. However, there were few studies to test the gas permeability of 3D lung porous scaffolds [26], although the mature methods for permeability testing in the food packaging field have already been established [17]. In this study, we designed a facile device to evaluate gas permeability of porous scaffolds for lung tissue engineering with a syringe pump as the gas transportation component and detergent film. According to Levy's research, the bubble meter method showed an accuracy of 99.75% in the measurement [18]. We conducted three sets of experiments to confirm the feasibility and accuracy of our device. The testing results without scaffolds showed the linear relation of the gas flow rate and the movement rate of the detergent film. Then, the scaffolds' pore morphology and materials on gas permeability were analyzed.

We fabricated PLLA salt leaching porous scaffolds, PU salt leaching porous scaffolds, and PU inverse opal scaffolds with different pore sizes for gas permeability tests. It has been reported that various techniques including solvent casting/particulate leaching, gas foaming, and phase separation technology could produce porous scaffolds (with porosities of ~35-60%) but with limited interconnectivity between pores [28]. Zhang et al. demonstrated that inverse opal scaffolds (porosity of ~74%) with higher interconnectivity (window size of 60-70 *μ*m) had a higher diffusion rate of macromolecules (FITI-dextran, Mw ≈ 20k), in comparison to scaffolds with nonuniform pores and a window size of 30 *μ*m [29]. Our results of gas permeability were consistent with these reports. With a higher pore connection rate, PU inverse opal scaffolds had higher permeability than PU salt leaching scaffolds, which indicated the reliability of our device for gas permeability testing.

Next, we compared the materials' effect on gas permeability. We found that the PU salt leaching scaffolds had better gas permeability than PLLA scaffolds produced by the same technique, which demonstrated that PU has a better gas permeability property and is much more suitable for lung tissue engineering in comparison to PLLA. Bao et al. reported that the O_2_ and CO_2_ permeation of PLLA used for packaging was 0.14 Barrers (at 22.8°C) and 1.1 Barrers (at 30°C), respectively [30], while the O_2_ and CO_2_ permeation of PU reported by Turan and colleagues was 5.17 Barrers and 78.69 Barrers at 25°C, respectively [31]. Overall, considering our gas permeability results and previous studies, we conclude that the gas permeability of scaffolds was determined not only by the fabrication methods but also by the characteristics of the materials used. Our new gas permeability device accurately detected the individual and combinational factors for the gas permeability.

The applied flow rate of gas can also affect the detection results. We set up the flow rate as 5, 10, 20, 30, 40, and 50 mL/min to observe the floater's movement and noticed that it was hard to distinguish the permeability property of scaffolds when the rate was lower than 20 mL/min. The lower gas flow rate might not generate sufficient pressure. Because of the delicate detergent bubble, the flow rate cannot set higher than 50 mL/min as this would have led to the breaking of the detergent film. Therefore, the gas flow rate set at 30 or 40 mL/min was suitable for testing permeability of the porous scaffolds. This is a limitation of the new device. A more durable and sensitive floater needs to be discovered.

## 5. Conclusions

In the current work, we presented a useful strategy for fabrication of scaffolds for lung tissue engineering and designed a gas permeability tester to assist the fabrication. The polyurethane scaffolds produced by the inverse opal technique resulted in highly connective pores and elastic property and demonstrated high cellular attachment and colocalization of epithelial and endothelial cells on them into alveolus-like tissues. VEGF-modified scaffolds enhanced the outcomes. The designed gas permeability tester with the detergent film floater can distinguish the gas permeability in scaffolds with different pore morphologies and made from different materials.

## Figures and Tables

**Table 1:** Experimental parameters to control different diameters of gelatin particles.

Diameter of gelatin particles (*μ*m)	Flow rate of toluene solution (mL/h)	Flow rate of gelatin solution (mL/h)	Diameter of capillary (mm)
180 ± 18	24	4.8	0.5
250 ± 25	24	3.6	1
310 ± 45	24	6	0.5
430 ± 51	24	6	1

## Data Availability

All study data are included in the article.
